# Building Death Literacy and Social Capital Through Experiential Learning in Rural Communities

**DOI:** 10.1093/geroni/igaf122.2098

**Published:** 2025-12-31

**Authors:** Leah Rohlfsen

**Affiliations:** St. Lawrence University, Canton, New York, United States

## Abstract

According to Noonan et al. (2016), the four facets of ‘death literacy’ are knowledge, skills, experiential learning, and social action. This presentation will highlight a variety of opportunities available to undergraduate students at a liberal arts college in a rural community. Experiences like the CARE fellowships, letter exchanges, community-based placements at skilled nursing facilities, and a variety of relevant internships not only provide students the opportunity to achieve ‘death literacy,’ but each experience builds social capital within and beyond the local community. Within the local communities, this social capital contributes to a community’s capacity to develop and share knowledge, sustain caring support systems, and problem solve (Horsfall, Noonan, & Leonard, 2012). When students move to other communities and into a variety of occupations, social capital becomes a more widely distributed ‘public good’ that demonstrates ‘caring’ and can be used to serve other needs. This is particularly valuable given the desire for alternatives to medicalized dying and demographic changes in many rural communities. This presentation will highlight valuable commonalities in experiential learning opportunities such as regular contact with older adults and caregivers, strong mentors in the community, reflection and introspection on the experience, and peer support. Data from student experiences indicates that improved death literacy among young people contributes to building compassionate, caring communities that support those in old age and at the end-of-life. Indeed, when young people have more contact with older adults, they have lower ageism and less death anxiety (Barnett & Adams, 2018).

